# Gender representation in food and beverage print advertisements found in corner stores around schools in Peru and Guatemala

**DOI:** 10.1186/s13104-021-05812-4

**Published:** 2021-10-30

**Authors:** Sophia Mus, Lucila Rozas, Joaquin Barnoya, Peter Busse

**Affiliations:** 1 Departamento de Investigación, Unidad de Cirugía Cardiovascular de Guatemala, 8-00 9th Avenue, 11th zone, 01011 Guatemala City, Guatemala; 2 Grupo de Investigación en Comunicación y Salud, Instituto de Investigación Científica, Universidad de Lima, 4600 Javier Prado Este Avenue, Tower A, 11th Floor, 15023 Lima, Peru

**Keywords:** Marketing, Gender, Childhood, Advertisement, Print media

## Abstract

**Objective:**

The objective of this study is to assess gender representation in food and beverage print advertisements.

**Results:**

The study follows a quantitative descriptive approach. Using a content analysis technique, we assessed the gender representation in 200 food and beverage print advertisements found in corner stores located in four areas around schools in Lima, Peru, and Guatemala City, Guatemala (100 advertisements per country). A total of 36% of the print advertisements exhibited a male main character for the case of Guatemala, while in Peru 14% of the print advertisements presented a male main character. Furthermore, in Guatemala, 22% of the main characters were male animated characters. Moreover, 27% of the print advertisements in Guatemala and 17%, in Peru were visually male-oriented. Overall, male characters appeared alongside sports references and in varied settings, whereas female characters were usually holding or consuming the product. In conclusion, although the majority of variables used to assess the representation of gender in food and beverage print advertisements were gender-neutral, those showing gender representation were mostly male-oriented. Despite its limited findings, the study provides evidence for the formulation of public policies and educational content aimed to protect children’s and adolescents’ health from the effects of food marketing.

## Introduction

Childhood obesity has increased during the last few years, representing one of the most common chronic disease risk factors in adulthood, and a growing problem in the global public health agenda [1, 2]. Latin America faces a complex landscape, many countries (including Peru and Guatemala) struggle with a double burden of disease, with persistent malnutrition and increasing rates of overweight and obesity [3, 4]. In Peru, 37.3% and 22.7% of the population 15 years and older are overweight and obese, respectively [5]. In Guatemala, overweight and obesity rates among adolescents between 13–17 years of age are 28.8% and 7.7% respectively [6]. Food advertising is one of the factors contributing to the obesogenic environment and is considered an important policy area to halt the obesity epidemic [7, 8]. The food and beverage industry relies on several marketing strategies to influence children and adolescents’ preferences, including animated characters, celebrities, toy giveaways, and colors [9–13].

The food industry recognizes gender notions about food and eating, translating them into advertisements targeting males and females in specific ways. For example, young females tend to be more vulnerable to television food advertisements and food branding compared to young men [14, 15]. Research from high-income countries such as United Kingdom, United States, and Spain presents evidence on how advertisements in general, and food advertisements in particular, exhibit elements of gender stereotyping [16, 17]. Gender-specific messages in television advertisements influence differently children’s notions about eating and physical activity [18]. Yet, there is a lack of evidence regarding gender representation in food and beverage print advertisements, specifically from middle-income countries like Peru and Guatemala. Therefore, we aim to describe the representation of gender in food and beverage print advertisements collected from corner stores located near schools in Guatemala City and Lima.

## Main text

### Methods

We have explained the methods in detail elsewhere [19, 20] but we briefly describe them here. Researchers visited four areas (400-m radius each) around public and private schools in Lima, Peru, and Guatemala City, Guatemala. They located all of the corner stores within the area using mobile phones and a Global Positioning System (GPS) device. Based on food and beverage categories commonly advertised in corner stores [11, 21], they took pictures of all print advertisements found in the corner stores. The sample consisting of 200 unique food and beverage print advertisements (100 for each country) was defined according to a saturation criterion, because at some point during the fieldwork period (October 2018–December 2018), researchers could not find new print advertisements to photograph. Moreover, researchers photographed some print advertisements more than once in different fieldwork areas, which meant discarding duplicated images according to pre-established image quality criteria (focus, sharpness, clarity, and absence of unnecessary visual elements in the photograph). A complete set of criteria for capturing and selecting images for analysis is published as supporting documentation in an open data repository [19].

To analyze gender representation, we worked together with a marketing consultancy firm in Guatemala (DUO Marcas) [22]. Together, we identified variables for the analysis based on their expertise in gender advertising and added other variables from prior studies [23–26]: brand, product category, general communication concept, color, product representation, environment/featured activity, number, and gender of the persons shown in the ad, health claim, values/emotional appeal, gender of the main character, role model/ public figure, public to which it is visually directed, focal point, typography, scales, shape, and contrast. A document with the variable definitions, as well as a document specifying the specific values for coding, are available together with the datasets of the study that we published in an open data repository [19, 20].

### Results

Sugar-sweetened beverages (SSB) were the most commonly advertised product (51%), even more so in Guatemala (61%) than in Peru (41%). Of the SSB print advertisements, 25% belonged to energy drinks. In contrast, print advertisements for water were rare: 12% in Peru and 4% in Guatemala (Table 1).

**Table 1:** Product Categories in the 200 Evaluated Print Advertisements Found in Corner Stores Around Schools from Lima Perú, and Guatemala City, Guatemala (n = 200)

	n	%
Sugar-sweetened beverages	102	51
Candy	20	10
Dairy	19	9.5
Water	16	8
Salty Snacks	13	6.5
Ice cream	10	5
Bread and pastry	9	4.5
Instantly prepared food	4	2
Cereals	3	1.5
Others	4	2

Gender representation was not present or present in very low rates (less than 10% of the total number of 200 print advertisements) in the following variables: *brand, product category, general communication concept, color, environment/featured activity, health claim, values/emotional appeal, focal point, typography, scales, shape, and contrast*. In comparison, the variables number *and gender of the persons shown in the ad*, *gender of the main character*, *product representation*, and *public to which the ad is visually oriented* showed some gender representation in 15–35% of the total number of print advertisements.

For the variable *number and gender of persons in the ad*, print advertisements with no characters (77% in Guatemala and 81% from Peru) were classified as gender-neutral, whereas print advertisements with female representation (showing female characters predominantly) were 10% in each city. We found more male-oriented print advertisements for this variable in Guatemala (13%) than in Peru (9%) (Table 2).

**Table 2:** Gender Representation in Food and Beverage Print Advertisements in Corner Stores Around Schools from Lima, Perú, and Guatemala City, Guatemala

	Perú (n = 100)			Guatemala (n = 100)		
	Neutral n (%)	Female n (%)	Male n (%)	Neutral n (%)	Female n (%)	Male n (%)
Product representation	87 (87%)	5 (5%)	8 (8%)	78 (78%)	2 (2%)	20 (20%)
Number and gender of the persons shown in the ad	81 (81%)	10 (10%)	9 (9%)	77 (77%)	10 (10%)	13 (13%)
Gender of the main character	74 (74%)	12 (12%)	14 (14%)	52 (52%)	12 (12%)	36 (36%)
Public to which the ad is visually oriented	70 (70%)	13 (13%)	17 (17%)	60 (60%)	13 (13%)	27 (27%)

Row percentages per country add up to 100%

Nearly half (48%) of the print advertisements from Guatemala and 26% from Peru had a main character that was either animated (i.e., a cartoon character) or a real person. The presence of a male main character was higher in Guatemala (36%) than in Peru (14%). We found no difference between countries regarding the percentage of print advertisements with female main characters (Table 2). From the total of print advertisements that presented the main character, 28 ads from Guatemala had the presence of animated characters, most of which were male-oriented (22 of 28 ads). In these ads, we assigned male representation because the character displayed characteristics that are considered typically masculine, such as clothing, activities (i.e., male-dominated sports), physical characteristics, and personality (i.e., strength, attitude). In Peru, the presence of animated characters was less frequent (nine of the 26 print advertisements that presented the main character).

From the number of print advertisements with the main character, 17% presented human characters in advertisements from Peru and 20% from Guatemala. Male characters, usually athletes, appeared in SSB print advertisements (mainly energy drink and soda brands) doing sports-related activities. Female characters were also found in SSB print advertisements and other products (e.g., yogurt, oatmeal). These characters appeared generally outdoors and displaying or consuming the featured product. In Peru, only two print advertisements presented women doing sports or at work. Nine print advertisements from Guatemala had celebrities (actors or athletes) as main characters, five of which were male. In Peru, four print advertisements displayed celebrities as the main characters (all male).

We referred to the set of graphic elements present in print advertisements (e.g., character portrayal, typography, setting, color, and scale) to assess the gender representation of the variables *product representation* and the *public to which the ad is visually oriented.* Regarding product representation, 20% of the print advertisements in Guatemala and 8% in Peru were male-oriented, whereas female-oriented print advertisements were 2% and 5%, respectively (Table 2). The print advertisements were visually male-oriented in 27% of the print advertisements in Guatemala and 17% in Peru, while female orientation was 13% in each country (Table 2). In the male-oriented print advertisements, the character (human or animated) was depicted both in a photograph in the print advertisement and in the product’s package. Also, male-oriented print advertisements contained typographies, colors, and design elements that could be considered masculine due to their characteristics (i.e., bold, rudeness, sharpness). Women were frequently shown holding or consuming the advertised product.

## Discussion

Our study found a higher prevalence of male characters in the food and beverage print advertisements, with male animated characters appearing more frequently. This finding is consistent with previous research showing that male main characters were prevalent in food advertisements [23]. This is also relevant because, as evidence suggests, animated characters capture children´s attention and influence their perception of taste, especially at a young age [27, 28]. Furthermore, the gender of the main character in a product may influence the consumption of boys and girls differently, as they are more likely to choose a product advertised with a character of the same gender [29].

Male main characters were usually paired with sports references and in a variety of settings (i.e., music festivals, public places, academic contexts), appearing both in the ad and on the product package. Interestingly, one-quarter of the SSB advertisements depicted energy drinks and had a famous athlete as the main character. Sports characters have been found to influence boys' purchase behavior and using sports celebrities in food products seems to increase the perceptions of healthfulness of such products [30, 31].

Women represented in the print advertisements usually appeared holding or consuming the featured product, frequently SSB such as sodas and fruit powdered drinks, or “healthy” products, such as yogurt and oatmeal. The extent to which our findings might influence female consumption patterns is yet unknown. However, adolescent girls have been found to perceive a healthy woman as thin [32], which may indicate that using female characters could influence girls’ food purchasing behavior.

Research shows that food marketing influences children´s food behavior and preferences, especially for low-nutrition, energy-dense food products [33, 34]. The tobacco industry has relied on marketing strategies to target their products to different gender groups [24, 25, 35]. Nonetheless, studies on how the food industry uses gender-based marketing strategies to target consumers are scarce.

Our study found that, although the majority of variables used to assess the representation of gender in food and beverage print advertisements were gender-neutral, those showing some gender representation (*number and gender of the persons shown in the ad*, *gender of the main character*, *product representation* and *the public to which the ad is visually oriented*) were mostly male-oriented. Moreover, these elements tended to depict stereotypical gendered attributes, activities, and behaviors, which could reinforce different food consumption behaviors among children and adolescents.

## Limitations

The study has limitations as the sample was not representative of all food and beverage print advertisements found in corner stores in Peru and Guatemala, given that we only concentrated our data collection in two cities for a limited period. Besides, we did not assess the actual advertisements’ impact on consumption patterns based on gender. Moreover, the results show that gender representation in print advertisements is not frequent, so our conclusions are limited to the few advertisements that show some gender representation. However, this research represents the first attempt to analyze food and beverage print advertisements with a gender perspective in Latin America.

Further research should focus on identifying the impact of gender-oriented marketing techniques employed by the food industry, particularly those found in print media placed inside corner stores of low and middle-income countries.

This study offers important evidence for the design of public policies and educational content that aim to protect children’s and adolescents’ health from the effects of food marketing, as it shows how the food industry is using gender stereotypes to appeal to and persuade different consumers.

## Data Availability

The datasets generated and analyzed during the current study can be freely and openly accessed on the openICPSR repository under the reference number openicpsr-122441, https://doi.org/10.3886/E122441V4. Moreover, a data paper describing in detail the data and the methods employed to collect has been published in the journal BMC Research Notes and is also listed as a reference in the manuscript. Please see reference numbers [19] and [20] for details and links to the data.

## Abbreviations

GPS: Global positioning system
SSB: Sugar-sweetened beverages
